# Fetal Clinical and Paraclinical Outcomes in HIV-Positive Pregnant Women

**DOI:** 10.7759/cureus.59568

**Published:** 2024-05-03

**Authors:** Madalina Daniela Iordache, Daniela Catalina Meca, Monica Mihaela Cirstoiu

**Affiliations:** 1 Department of Obstetrics and Gynaecology, University Emergency Hospital Bucharest, Doctoral School of Carol Davila University of Medicine and Pharmacy, Bucharest, ROU; 2 Department of Obstetrics and Gynaecology, University Emergency Hospital Bucharest, Carol Davila University of Medicine and Pharmacy, Bucharest, ROU

**Keywords:** 1-min apgar score, blood count, preterm birth, low birth weight, human immunodeficiency virus, pregnancy

## Abstract

Background

Adverse pregnancy outcomes in women with human immunodeficiency virus (HIV) infection remain significantly increased. Untreated maternal infection primarily leads to fetal complications, such as intrauterine growth restriction, stillbirth, or preterm birth. Concerning both maternal and fetal complications that can appear in pregnancy associated with HIV infection, the purpose of the study was to determine fetal and maternal demographic characteristics and the correlation between blood count parameters and poor fetal prognosis.

Methods

We conducted a quantitative study utilizing document review as the data collection method. This study encompassed a cohort of nine HIV-positive pregnant women who delivered at the Obstetrics and Gynecology Department of the University Emergency Hospital in Bucharest from January 1, 2021, to December 31, 2023. A comparative cohort of nine healthy pregnant women who delivered during the same period in the same facility was selected using stratified random sampling. We examined maternal and fetal demographic parameters and neonatal outcomes, reporting them to paraclinical laboratory data.

Results

The incidence of pregnancy-related HIV infections was 0.16%. The mean age of patients in the selected group was 29.88 ± 5.53. There was no statistically significant correlation between maternal clinical and paraclinical parameters in the HIV-positive and HIV-negative groups. Although there was a slightly negative difference in the fetal weight at birth, the 1-min APGAR (appearance, pulse, grimace, activity, and respiration) score, and the intrauterine growth restriction between the two groups, there was a statistically significant association between admission to the neonatal intensive care unit (NICU) and the neonates from HIV-positive pregnancies. In our study, we observed preterm deliveries in 22.22% of cases, and we did not record any stillbirths. The 1-min APGAR score was correlated with the value of leukocytes in peripheral blood. Vertical transmission was established to be 11.11% independent of maternal blood count parameters.

Conclusion

HIV infection during pregnancy leads to a higher risk of admission to the NICU. Fetal leukocytosis is indicative of a lower 1-min APGAR score. The primary emphasis of therapeutic intervention during pregnancy should center on vigilant monitoring of maternal viral load and the timely administration of antiretroviral therapy to enhance fetal outcomes.

## Introduction

Human immunodeficiency virus (HIV) infection is a severe global health problem that leads to a progressive failure of the immune system. Its prevalence varies between high- and low-income countries, from 0.1-2/1000 patients to 29% [1,2]. Although the seroprevalence varies dramatically by geographic region, HIV infection during pregnancy has been reported in some studies to be 1.7 per 1000 pregnancies [3,4]. However, Drake et al.’s (2014) meta-analysis revealed that there was a slight difference between the incidence among pregnant women with HIV and the general population [5].

HIV infection during pregnancy is associated with increased fetal morbidity and mortality, especially in cases with a higher viral load and in the absence of antiretroviral treatment [6].

Wedi et al.’s (2016) systematic review and meta-analysis showed that HIV infection is associated with an increased risk of preterm birth, intrauterine growth restriction, and stillbirth [7]. The mechanism for preterm birth and subsequently for low-birth-weight neonates in HIV-positive pregnant women is unknown. However, it is thought to involve sociocultural factors and abnormal maternal vascular development, which is not correlated with reduced growth factors or decreased angiogenesis [8]. Another study reinforced the idea that HIV infection or antiretroviral therapy is associated with immune dysregulation in the placental unit, which increases the risk of preterm birth [9].

Excluding the risks of obstetrical adverse outcomes during pregnancy, HIV-positive pregnant women are at increased risk of anemia due to iron deficiency, systemic inflammation, and different infections [10]. Regarding the peripheral blood count, HIV-positive pregnant women are associated with an increased total white cell count. However, it was found that pan-lymphopenia and neutrophilia were not linked to HIV infection [11].

Taking into consideration the fetal outcome in terms of hematological measurements, it is stipulated that at birth, there is no difference between infants of HIV-positive and HIV-negative pregnant women, except for thrombocytopenia [12]. The management of HIV infection in fertile patients must include pre-conception counseling because there is an improved prognosis in patients with minimum viral load. Many studies do not show any risk of HIV progression during pregnancy [13]. The vertical transmission rate is 20-25% for HIV-1 and 5% for HIV-2 [14]. Scheduled cesarean delivery and antiretroviral therapy are modalities to decrease vertical transmission [15].

## Materials and methods

We conducted a descriptive, retrospective study that included nine HIV-positive pregnant women who delivered in the Obstetrics and Gynecology Department of University Emergency Hospital in Bucharest between January 1, 2021, and December 31, 2023, and a control group of nine pregnant women with similar age and without any pathologies who delivered in our unit during the same period. The University Emergency Hospital’s database system was used to collect the maternal and fetal clinical and paraclinical data. All the patients in the study signed the informed consent.

The inclusion criteria were as follows. For the study group: age over 18 years, the presence of informed consent, and all pregnant women known to be HIV-positive or who were diagnosed during their respective hospitalizations. The control group’s inclusion criterion was represented by a negative HIV test at admission. Control group patients were selected randomly, but the maternal age and the absence of any systemic pathology (e.g., thrombophilia, autoimmune diseases)/pathologies associated with pregnancy (e.g., pregnancy-induced hypertension, gestational diabetes)/bacterial or viral infection were considered.

Approval for the study was granted by the Ethical Committee of the University Emergency Hospital in Bucharest (Np.58134/16.11.2020).

Data analysis was performed using SPSS 26.0 software. The Pearson correlation test was used to establish the correlation between fetal outcome (gestational age at delivery, fetal weight, 1-minute APGAR (appearance, pulse, grimace, activity, and respiration) score, admission to NICU), and paraclinical maternal parameters. All the results considered statistically significant had p < 0.05.

## Results

Out of 5,486 births registered in the University Emergency Hospital during the three years of our study, only nine patients were HIV-positive and eligible for our research. The incidence of HIV infection associated with pregnancy was 0.16% over this period.

The patients in the control group were selected randomly, specifying that the fetal outcomes were not considered. All the pregnancies were singletons, as in the HIV-positive group, and none of the pregnant women had systemic pathology or pregnancy complications (pregnancy-induced hypertension, gestational diabetes, bacterial infection).

The maternal and fetal demographic and clinical features are described in Table 1.

**Table 1:** Demographic and Clinical Features of the Groups Under Study

		HIV Group	Control Group	p-value
Maternal demographic data				
Maternal age, years (mean ± SD)		29.88 ± 5.53	29.44 ± 6.72	0.826
BMI (mean ± SD)		21.47 ± 1.60	24.28 ± 2.41	0.007
Area of residence	Urban N (%)	8 (88.89%)	6 (66.67%)	0.170
	Rural N (%)	1 (11.11%)	3 (33.33%)	
Investigated pregnancy N (%)		3 (33.33%)	8 (88.89%)	0.013
Days of hospitalization (mean ± SD)		6.1 ± 4.72	3.77 ± 0.83	0.360
Hepatitis C N (%)		3 (33.33%)	0	-
Hepatitis B N (%)		1 (11.11%)	0	
Genito-urinary infection N (%)		2 (22.22%)	0	
Drug addict N (%)		2 (22.22%)	0	
Fetal outcomes				
Birth weight (grams) (mean ± SD)		2870 ± 495.94	3142.22 ± 370.87	0.308
1-minute APGAR score (mean ± SD)		7.9 ± 1.28	8.33 ± 0.87	0.312
Intrauterine growth restriction N (%)		2 (22.22%)	1 (11.11%)	0.347
NICU admission N (%)		5 (55.56%)	1 (11.11%)	0.035
Gestational age (weeks) (mean ± SD)		37.74 ± 1.87	38.44 ± 1.50	0.942

It should be noted that the p-value is statistically significant regarding medical care during pregnancy. However, in the HIV group, we can specify that the hospital admission period was prolonged compared with the control group (6.1 ± 4.72 days vs. 3.77 ± 0.83 days). We noticed that 33.33% of the cases were positive for hepatitis C (HCV) and 11.11% for hepatitis B (HBV) in the HIV group. Genito-urinary infections were found in two cases (22.22%), both involving *Escherichia coli*. In addition, the medical history of the patients revealed one case with an ischemic stroke and one case with polymorphic psychotic disorder. All of the HIV group’s patients had normal liver and renal functions.

Taking into consideration the living conditions, two of the pregnant women in the HIV group were drug addicts.

None of our study’s patients received specialized antiretroviral treatment during pregnancy. The reasons were the lack of periodic medical check-ups in most cases or the absence of viremia. The HIV combi assay was performed in only five cases, with a mean value of 292.58 S/CO (95% CI, 130.029-455.131). There was no statistically significant correlation between the results of the HIV combi assay and the fetal outcomes (birth weight p = 0.481, 1-min APGAR score p = 0.06).

The mode of delivery in the HIV group was an emergency cesarean section in 77.78% (seven cases). The control group registered the same percentage of spontaneous births. The leading indicators for emergency cesarean section in our study group were the prevention of vertical transmission of HIV infection and the imminent risk of uterine rupture (in five cases, the patients had undergone a cesarean section in the past). Eight of nine patients had a favorable evolution after birth, while one underwent a necessary hysterectomy.

**Figure 1:**
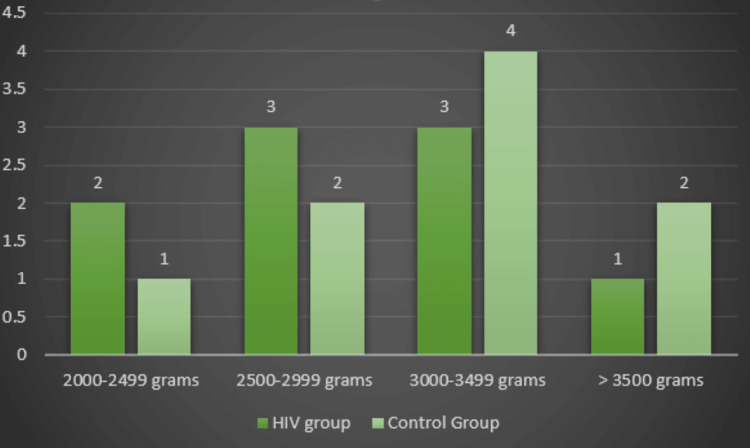
The distribution of fetal weight in the groups under study

**Figure 2:**
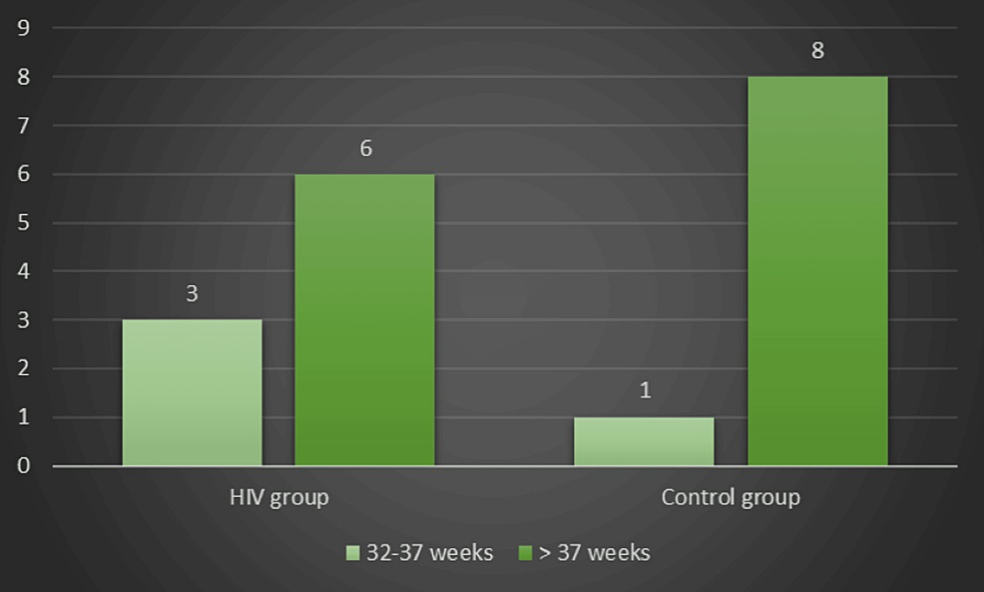
The distribution of preterm birth in the groups under study

We found a statistically significant difference between the groups regarding NICU admission when we examined the fetal outcomes in the HIV group. The birthweight was higher in the control group (2870 ± 495.94 g vs. 3142.22 ± 370.87 g), indicating no cases of extremely or very premature births in both groups (Figures 1-2). No stillbirths were registered in our study group.

All newborns received prophylactic antiretroviral therapy (lamivudine and zidovudine). However, in 88.89% of the cases, the HIV combi PT assay was negative. The fetal HIV combi assay was positive (143.08 S/CO) in one case but without any correlation between the fetal and maternal viral results. Therefore, there was no direct correlation between those test results and the risk of vertical transmission; the mean value of HIV combi PT assay in the cases with negative fetal tests at birth was 314.53 ± 206.51 S/Co, showing that in the case with positive fetal combi assay result, the maternal test did not reveal a higher value than in the cases with negative fetal test.

We established no statistically significant correlation between the blood count parameters in the two groups regarding the laboratory analysis (Table 2). Analyzing the fetal blood count in the HIV group revealed that there was a statistically significant correlation between hemoglobin, hematocrit, leukocyte, lymphocyte, eosinophil, and basophil values relative to the maternal blood count parameters (p <0.05) (Tables 2-3).

**Table 2:** Maternal blood count parameters in the groups under study

	Maternal Blood Count in HIV Group	Maternal Blood Count in Control Group	p-value
RBC 10*6/uL (mean ± SD)	4.01 ± 0.49	3.64 ± 0.47	0.867
Hemoglobin g/dL	10.74 ±0.89	11.36 ± 1.17	0.926
Hematocrit %	33.09 ±2.78	32.86 ± 3.61	0.872
Platelets 10*3/uL	200.33 ± 91.14	261.56 ± 87.39	0.527
Neutrophils %	77.74 ±10.06	74.96 ± 6.21	0.853
Leukocytes %	10.06 ±2.96	12.46 ± 3.45	0.918
Lymphocytes %	14.87 ±6.97	14.41 ± 8.60	0.393
Monocytes %	5.36 ± 2.36	5.67 ± 1.53	0.683
Basophils %	0.49 ± 0.18	0.49 ± 0.26	0.890
Eosinophils %	0.19 ± 0.52	0.42 ± 0.48	0.665

**Table 3:** Maternal and fetal blood count distribution in the HIV group

	Maternal Blood Count in HIV Group	Fetal Blood Count in HIV Group	p-value
RBC 10*6/uL (mean ± SD)	4.01 ± 0.49	4.51 ± 0.40	0.762
Hemoglobin g/dL	10.74 ±0.89	16.49 ± 1.78	
Hematocrit %	33.09 ±2.78	48.7 ± 5.63	
Platelets 10*3/uL	200.33 ± 91.14	260.22 ±70.76	0.065
Neutrophils %	77.74 ±10.06	58.26 ± 7.43	0.960
Leukocytes %	10.06 ±2.96	16 ± 6.16	0.042
Lymphocytes %	14.87 ±6.97	27.52 ±6.26	0.002
Monocytes %	5.36 ± 2.36	9.57 ± 3.54	0.211
Basophils %	0.49 ± 0.18	1.04 ± 0.27	0.002
Eosinophils %	0.19 ± 0.52	2.52 ± 1.75	0.010

**Figure 3:**
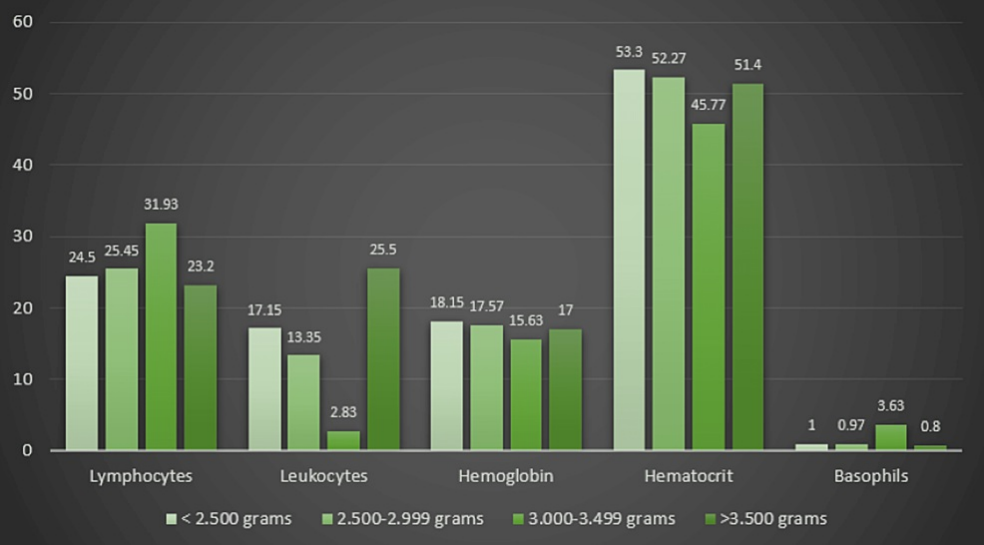
Distribution of fetal blood parameters in correlation with the birthweight in the HIV Group

**Figure 4:**
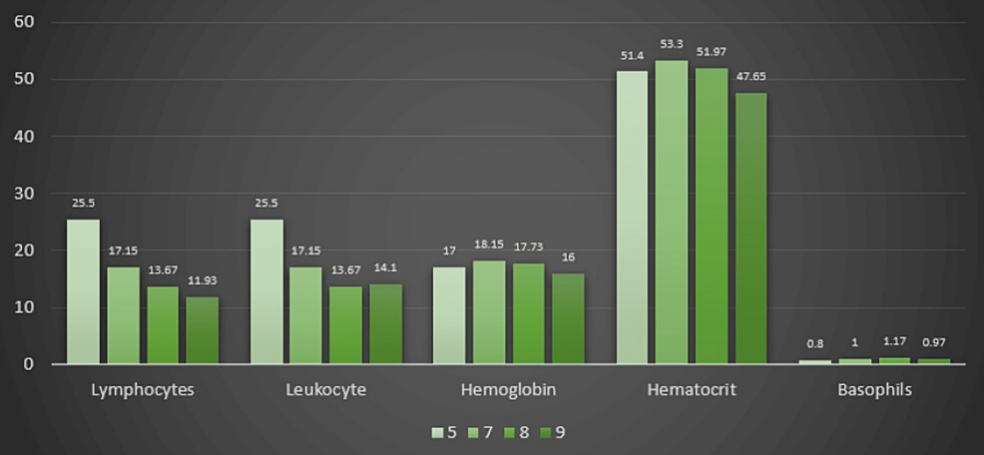
Distribution of fetal blood parameters in correlation with the 1-min APGAR Score in the HIV Group

Considering these features of the blood count, we learned that there was no significant correlation between preterm birth and fetal blood parameters (Figure 3). The 1-min APGAR score indicated a positive correlation between the leukocyte values and the APGAR score. Therefore, lower values of this blood count parameter are correlated with a higher 1-min APGAR score (Figure 4).

## Discussion

In the absence of specialized treatment, HIV infection is a life-threatening pathology that has spread worldwide. The World Health Organization notes an incidence of 0.7% in adults aged 15-49 years old, with significant variations between countries and regions [16].

In our study, the incidence of HIV infection during pregnancy was 0.16% in a group of 5,486 pregnant women, similar to that described in Kurtay and Hussein’s (2022) study on a group of 7,959 pregnant women (0.2%) [17]. In our research, the mean age of HIV-positive pregnant women was similar to that of the control group (29.88 ± 5.53 vs. 29.44 ± 6.72 years). An age under 30 was associated with an improved pregnancy prognosis in Wu et al.’s (2023) study [18]. The BMI represented the significative demographic feature, with lower values in the HIV-positive group (p = 0.007). This characteristic contradicts the findings in Kwiatkowska et al.’s (2013) study, which revealed no differences between the BMI values in HIV-positive or negative women due to a high risk of central obesity in HIV-positive women [19].

A study conducted by Ørbæk et al. (2020) reported the following risk factors in HIV-positive pregnant women: smoking, chronic HBV and HCV, psychiatric disorders, or a prior cesarean section [20]. In our study, seven of nine cases were smokers (77.89%), while drug addiction was observed in 22.22% of the cases. Psychiatric pathology was found in 11.11% of the cases, while chronic HCV was detected in 33.33% of the cases. A prior cesarean section was observed in 55.56% of the cases. No perinatal death was registered in the patients’ obstetrical history.

A meta-analysis of cohort studies published by Xiao et al. (2015) showed that there was no significant difference between pregnancy outcomes and HIV infection. However, there is a significant heterogeneity among studies regarding the risk for low birth weight and preterm birth [21]. The European Foundation for the Care of Newborn Infants notes a higher risk for preterm delivery in the HIV-positive group compared with HIV-negative pregnant women (31.6% vs. 13.5%) [22].

Yingjuan et al.’s (2023) retrospective cohort study indicated a 13.51% risk of preterm birth in an HIV-positive group, compared with 6.82% in the HIV-negative group. In addition, the risk for low birth weight was significantly higher in the HIV-positive group (14.17% vs. 4.65%) [23]. A study performed by Dadhwal et al. (2017) regarding maternal and fetal outcomes in HIV-positive pregnant women highlighted that, although there were no statistically significant results, there was a significant number of cases with intrauterine growth restriction compared to a group of HIV-negative pregnancies [24]. Our study showed a 22.22% risk of preterm birth in the HIV-positive group; all the cases involved were categorized as moderate to late preterm (32-37 weeks of pregnancy). This percentage is between the values established in other studies, considering that the findings were not statistically significant. The risk for low weight at birth was higher in the HIV group but without a statistically significant correlation. Only 22.22% of newborns had a birth weight below 2.500 grams, and intrauterine growth restriction was observed at the same percentage.

In our study, the 1-minute APGAR score was insignificantly lower in the HIV-positive group compared to the control group (7.9 ± 1.28 vs. 8.33 ± 0.87). It is important to mention that 55.56% of newborns in our study group needed admission to the NICU, in two cases because of prematurity. In the other cases, a transient tachypnea was involved. This complication was also mentioned in Kreitchmann et al.’s (2011) study regarding neonatal respiratory morbidity in infants exposed to HIV infection [25].

Our study did not reveal an increased risk for anemia or leukocytosis in the HIV-positive group regarding maternal paraclinical features. The statistically insignificant correlation between maternal blood count and HIV infection (p = 0.47) was also reported in Dadhwal’s (2017) study [24]. Methazia et al.’s (2020) research contradicts these findings, stating that moderate or severe anemia is expected to occur more often in HIV-positive pregnant women, worsening the outcomes [26]. Although HIV-positive status represents a higher risk for puerperal sepsis and cesarean section, as mentioned in other studies, in our research, none of the patients developed infectious complications [27]. However, the risk for cesarean section was higher than in the control group (77.78% vs 22.22%).

The maternal distribution of white blood count in both groups reveals that being HIV-positive has no influence on leukocyte and lymphocyte subsets. Furthermore, this data is bolstered by Mandala et al.’s (2017) study, in which the CD4+ results are also described [11].

Regarding the fetal blood count in the HIV group, we discovered a directly proportional, statistically significant relationship between maternal and fetal parameters regarding hemoglobin, hematocrit, leukocytes, basophils, and eosinophils. However, the blood count parameters did not influence the fetal weight, the risk for intrauterine growth restriction, or admission to the NICU. A statistically significant relationship was established between the 1-min APGAR score and the leukocyte values in fetal blood.

Study limitations

The primary limitation of this study is the small sample size caused by the low incidence of HIV, due to diligent interventions, control, and prevention of HIV infection worldwide. The lack of supplementary laboratory analysis necessary for an HIV-positive patient (CD4 cell counts, viral load measurement) is another limitation of the study. Those missing data overlapping on a small sample size hinders understanding of the severity of HIV infection in the participants. A further limitation of the study is the lack of neonatal medical check-ups after discharge, including testing for HIV-RNA. Also, positive women were redirected to infectious diseases hospitals to receive adequate treatment, without being able to have medical information about their subsequent course. The Prevention of Mother-to-Child Transmission (PMTCT) program recommends follow-ups and check-ups for HIV PCR and antibody tests before two years of age. 75% of infants who were HIV-negative at birth will become positive after seven days, suggesting an intrapartum infection, while 90% of infected infants will test positive after 14 days [28].

## Conclusions

Despite being derived from a limited sample size, the study demonstrated elevated fetal morbidity and mortality, as evidenced by a higher proportion of neonates requiring intensive care treatment (p=0.035). A significant negative correlation was observed between the 1-minute APGAR score and leukocytosis in fetal peripheral blood (p=0.009). Factors such as intrauterine growth restriction, preterm birth, and the vertical transmission of HIV significantly influence fetal prognosis. However, the administration of HIV treatment prior to and during pregnancy has been shown to mitigate fetal complications.
